# Comparative Case Study of Two Biomedical Research Collaboratories

**DOI:** 10.2196/jmir.7.5.e53

**Published:** 2005-10-25

**Authors:** Titus KL Schleyer, Stephanie D Teasley, Rishi Bhatnagar

**Affiliations:** ^3^True Commerce IncWexfordPAUSA; ^2^Collaboratory for Research on Electronic WorkSchool of InformationUniversity of MichiganAnn ArborMIUSA; ^1^Center for Dental InformaticsSchool of Dental MedicineUniversity of PittsburghPittsburghPAUSA

**Keywords:** Biomedical research, collaboration, collaboratories, community networks, information technology, interdisciplinary research

## Abstract

**Background:**

Working together efficiently and effectively presents a significant challenge in large-scale, complex, interdisciplinary research projects. Collaboratories are a nascent method to help meet this challenge. However, formal collaboratories in biomedical research centers are the exception rather than the rule.

**Objective:**

The main purpose of this paper is to compare and describe two collaboratories that used off-the-shelf tools and relatively modest resources to support the scientific activity of two biomedical research centers. The two centers were the Great Lakes Regional Center for AIDS Research (HIV/AIDS Center) and the New York University Oral Cancer Research for Adolescent and Adult Health Promotion Center (Oral Cancer Center).

**Methods:**

In each collaboratory, we used semistructured interviews, surveys, and contextual inquiry to assess user needs and define the technology requirements. We evaluated and selected commercial software applications by comparing their feature sets with requirements and then pilot-testing the applications. Local and remote support staff cooperated in the implementation and end user training for the collaborative tools. Collaboratory staff evaluated each implementation by analyzing utilization data, administering user surveys, and functioning as participant observers.

**Results:**

The HIV/AIDS Center primarily required real-time interaction for developing projects and attracting new participants to the center; the Oral Cancer Center, on the other hand, mainly needed tools to support distributed and asynchronous work in small research groups. The HIV/AIDS Center’s collaboratory included a center-wide website that also served as the launch point for collaboratory applications, such as NetMeeting, Timbuktu Conference, PlaceWare Auditorium, and iVisit. The collaboratory of the Oral Cancer Center used Groove and Genesys Web conferencing. The HIV/AIDS Center was successful in attracting new scientists to HIV/AIDS research, and members used the collaboratory for developing and implementing new research studies. The Oral Cancer Center successfully supported highly distributed and asynchronous research, and the collaboratory facilitated real-time interaction for analyzing data and preparing publications.

**Conclusions:**

The two collaboratory implementations demonstrated the feasibility of supporting biomedical research centers using off-the-shelf commercial tools, but they also identified several barriers to successful collaboration. These barriers included computing platform incompatibilities, network infrastructure complexity, variable availability of local versus remote IT support, low computer and collaborative software literacy, and insufficient maturity of available collaborative software. Factors enabling collaboratory use included collaboration incentives through funding mechanism, a collaborative versus competitive relationship of researchers, leadership by example, and tools well matched to tasks and technical progress. Integrating electronic collaborative tools into routine scientific practice can be successful but requires further research on the technical, social, and behavioral factors influencing the adoption and use of collaboratories.

## Introduction

Collaborating across geographic and disciplinary boundaries presents research initiatives with an unprecedented challenge in terms of communication and collaboration [1-3]. Meeting these challenges successfully requires unconventional tools and novel scientific work practices [4,5]. The “collaboratory” [6-8] has emerged as a concept for an infrastructure that supports new methods for collaboration using electronic communication networks. In a National Science Foundation workshop held in 1989, William Wulf proposed that “integrated, tool-oriented computing and communications systems to support scientific collaboration...can be called ‘collaboratories.’ Collaboratories [are]...centers without walls, in which the nation's researchers can perform their research without regard to geographical location, interacting with colleagues, accessing instrumentation, sharing data and computational resources, and accessing information in digital libraries” [7].

Since the concept was initially proposed, collaboratories have become more widely known and adopted in biomedical research [9,10]. Examples of current and past biomedical research and development projects that included formal collaboratories include the following:

- the Biomedical Informatics Research Network (BIRN), which enables data sharing across neuroimaging databases throughout the United States [11]
- the Biological Collaborative Environment (BioCoRE), which is a collaborative research environment for molecular modeling and simulation [12]
- the Molecular Modeling Collaboratory, which is centered around the development, deployment, and use of a highly extensible, interactive molecular modeling software [13]
- the National Laboratory for the Study of Rural Telemedicine, which established the Virtual Hospital, a digital health sciences library and multimedia information integrator providing just-in-time access to information for medical practice, continuing education, and patient education [14]
- the Visible Human Project (VHP), which created complete, anatomically detailed, three-dimensional representations of the normal male and female human bodies [15]

In this paper, we report on the implementation and evaluation of two collaboratories funded by the National Institutes of Health (NIH) that were created to support distributed research centers in biomedicine. We use the term “center” to refer to the center grant as a whole, and the term “collaboratory” to denote the electronic infrastructure that supports communication and collaboration within each center. In contrast to the projects referenced above, most biomedical research centers do not include a formal collaboratory. Many center directors, being unfamiliar with electronic collaborative tools, simply expect traditional methods, such as phone, fax, email, and occasional face-to-face meetings, to support effective and efficient work toward the project objectives. While centers using more traditional communication methods reduce the technical complexity of operating the center, opportunities for more efficient, effective, and novel collaboration through new electronic tools are lost.

The main purpose of this paper is to evaluate comparatively two collaboratories that used off-the-shelf tools and relatively modest resources to support the scientific activity of two biomedical research centers. We first describe the two centers, their goals, and their institutional participants and personnel. Next, we discuss the requirements for collaboration and communication within each center and how we supported these requirements using commercially available electronic tools. Finally, we present selected utilization and outcomes data and conclude with a discussion of barriers and enablers that affected the technology adoption within each collaboratory.

The main goal of this report is to help stakeholders in geographically distributed research centers understand the potential applications of a collaboratory and how to implement one using off-the-shelf tools. Two secondary goals are (1) to provide collaboratory architects with guidance on requirements definition, tool selection, implementation, and evaluation, and (2) to contribute to the growing literature on collaboratory design and implementation [16-18].

 The collaboratories we describe were funded as part of the Great Lakes Regional Center for AIDS Research (HIV/AIDS Center) and the New York University (NYU) Oral Cancer Research for Adolescent and Adult Health Promotion Center (Oral Cancer Center). Both centers were large-scale, cooperative research projects funded by the NIH that focused on a single, complex, biomedical research problem. The HIV/AIDS Center, which is no longer operating, focused on HIV biology, immunology, vaccines, therapeutic trials, and behavioral science, and it included four academic institutions in the Midwestern United States. The Oral Cancer Center is currently addressing the reduction of health disparities in oral cancer and encompasses ten institutions. Table 1 provides an overview of both research projects.

**Table 1:** Summary of the Great Lakes Regional Center for AIDS Research (HIV/AIDS Center) and the NYU Oral Cancer Research for Adolescent and Adult Health Promotion Center (Oral Cancer Center)

**Center**	**HIV/AIDS Center**	**Oral Cancer Center**
Research topic	HIV/AIDS	Health disparities in oral cancer
Major organizational components	7 research areas 8 cores (administrative, clinical research, nonhuman primate model, genomics and proteomics, single-cell imaging and analysis, immunology resource, biocomplexity, and collaboratory)	4 research studies 3 cores (administrative, biostatistics, and collaboratory)
Research studies	HIV molecular biology HIV/AIDS pathogenesis research Epidemiology and natural history Opportunistic infections and AIDS-related malignancies Vaccine and other prevention research and development Therapeutic research and development Disease manifestations and metabolic complications	Risk factors for oral epithelial dysplasia Oral cancer detection: current and emerging technologies Cancer screening and research subject participation by minorities Personalized risk feedback in dental clinic smokers
Number of		
principal investigators (PIs)	12 (cores)	7 (4 studies and 3 cores; one study and one core are directed by the same PI)
research personnel (including PIs)	117	15
administrative personnel	4	9
Participating institutions	Northwestern University University of Michigan University of Minnesota University of Wisconsin	Boston University Howard University Johns Hopkins University Memorial Sloan-Kettering Cancer Center New York University Puerto Rico Health Department Tuskegee University University of Alabama/Birmingham University of Pittsburgh University of Puerto Rico
Project duration	9/1998 to 8/2003	8/2001 to 7/2008
Budget	$6.75 million (including $559000 for collaboratory)	$8.3 million (including $604000 for collaboratory)
Funded by	National Cancer Institute and National Institute of Allergy and Infectious Disease	National Institute of Dental and Craniofacial Research

The HIV/AIDS Center was comprised of eight cores engaged in seven research programs. The center’s mission was to promote multidisciplinary AIDS research and to engage more scientists in developing more effective measures to prevent and treat HIV infection. Based on several proposed research areas, the center created an infrastructure in which new projects were developed and supported. The program was originally funded for four years (1998-2002) and received an additional year of bridging funds in 2003. Competitive renewal applications were unsuccessful, leading to the dissolution of the center in September 2003.

In contrast to the more developmental focus of the HIV/AIDS Center, the Oral Cancer Center clearly defined four research studies prior to the start of the project; a fifth study will be developed later in the project period. Each of the study proposals clearly framed research questions and methods and described participating research personnel, infrastructure, and budgets. The four research studies are supported by the administrative, biostatistics, and collaboratory cores. While some of those institutions are geographically close (such as the Memorial Sloan-Kettering Cancer Center and NYU), others are quite remote (such as the University of Puerto Rico). The project is funded until 2008.

Both centers had a similar governance structure. They were administered by a lead institution (Northwestern University for the HIV/AIDS Center and NYU for the Oral Cancer Center) and were guided and managed by the group of principal investigators. Each center was advised by an external advisory committee composed of leading scientists in the field and a representative of the funding agency. The principal investigators were responsible for the day-to-day operations of their respective projects.

Unlike most research centers, both the HIV/AIDS Center and the Oral Cancer Center proposed a formal collaboratory in their grant application. The principal investigator for the HIV/AIDS Collaboratory was S. Teasley (University of Michigan), and for the Oral Cancer Collaboratory, T. Schleyer (University of Pittsburgh). Both individuals participated in their respective centers as full members of the scientific and administrative leadership.

In summary, the centers resembled each other in the following ways: their multidisciplinary approach to a single, complex research question; the involvement of several geographically distributed institutional participants; the inclusion of a dedicated collaboratory core; the size of the budget; and the length of the funding period. The major difference was that the HIV/AIDS Center built a platform to develop projects, while the Oral Cancer Center is focusing on the completion of predefined projects.

## Methods

### Needs Assessment and Initial Requirements Definition

Both collaboratories were developed by conducting a needs assessment and defining requirements; researching, evaluating and selecting off-the-shelf collaboration tools; creating custom resources, such as websites, when needed; and implementing and evaluating the collaboratory. To understand the specific needs of investigators and projects, the collaboratory staff in each center reviewed the grant application in detail and interviewed each principal investigator and key research personnel. These semistructured interviews addressed questions about tasks related to projects, interaction between project teams and center members, the project-related information generated and/or managed, and other center characteristics. In addition, we assessed the local computing infrastructure as well as the software applications used by each investigator, both for desktop computers and personal digital assistants. In both centers, we also conducted contextual inquiry [19] sessions with selected personnel.

### Tool Evaluation and Implementation

Once the requirements for a collaborative activity were sufficiently defined, technical staff researched and evaluated existing tools. This typically involved compiling lists of commercial and open-source applications, matching product features against requirements, and testing selected products. 

Once a product that satisfied a set of requirements was found, we implemented it in a pilot installation with selected center members. This approach allowed us to address most implementation and functionality issues before a large-scale rollout. Typically, the technical staff of each collaboratory worked with remote technical support to install and configure the tools on scientists’ desktops, conduct site-to-site pilot sessions or tests to ensure smooth functioning, and to train the research personnel. In this phase, technical staff members used collaborative tools, such as Timbuktu Pro (Netopia, Inc., Emeryville, CA, USA) and NetMeeting and Remote Desktop Connection (both Microsoft, Inc., Redmond, WA, USA). Scientists were given enough practice and on-site technical support to become comfortable with each tool as it was rolled out.

### Collaboratory Evaluation

The evaluation of the collaboratory implementation used three main approaches. First, we collected utilization data through manual and automatic data collection methods (such as Web logs). For example, for Web conferencing, we tracked parameters such as the number of sessions, participants, and participating computers; for collaborative applications, the utilization data included the number of participants, their usage of tools (such as calendars and meeting tools), and the number of shared files. Second, we conducted brief surveys to assess participants’ experience with and attitudes toward the tools. Third, we evaluated each collaboratory as participant observers [20] and engaged in repeated discussions with principal investigators and other research personnel, gaining valuable contextual data. As a final step in the evaluation, we identified barriers and enablers that affected the outcomes of the respective collaboratory implementations. The behavioral research within the HIV/AIDS Center was approved by the University of Michigan (IRB Protocol #B03-00001782) and, within the Oral Cancer Center, by the University of Pittsburgh (IRB Protocols #020722 and #0309076).

## Results

### Needs Assessment and Initial Requirements Definition

None of the investigators, research staff, or administrators in either center had experience with collaborative tools except email and locally shared data stores. Some individuals had participated in videoconferences, typically using PolyCom (Polycom Inc., Pleasanton, CA, USA). Therefore, early in Year 1 in both centers, we conducted educational sessions for the principal investigators and key research staff on the collaboratory concept and corresponding tools.

Within the HIV/AIDS Center, the needs assessment identified two activities as most important for supporting existing collaborations and getting new collaborations started. First, the scientists needed to run distributed lab meetings that would allow conversation over shared data, including, for example, images from a specialized microscope located at only one of the sites. The expectation for this activity was that it be fully interactive so that participants, from few to many, could interact with each other in real time. The importance of high-quality, real-time interactions has been shown to be important for scientific research [21]. Second, the scientists wanted a way to broadcast seminars to share information from experts inside and outside the center.

The needs assessment of the Oral Cancer Center suggested that the requirements for its collaboratory were different. One main objective was to facilitate interaction and tasks among the center participants at large, mostly from an administrative perspective. The second objective was to support the work in each project group. In this case, the requirements centered on facilitating small group communication; sharing protocols, raw research data, and analyses; and aiding workflow. The four research projects, however, differed significantly in their goals and objectives, operations, and personnel roles within the groups. For instance, in the research project on cancer screening and research subject participation by minorities, the initial work was highly sequenced and was either performed by one or two individuals at a time or by a group of research personnel (such as telephone interviewers) who required no support with collaborative tools. By contrast, the research project on personalized risk feedback in dental clinic smokers was highly interactive and data intensive. In this project, the research personnel at Memorial Sloan-Kettering Cancer Center (MSKCC) (who designed the study and analyzed the data) and the clinical personnel at NYU (who handled all patient interactions) interacted frequently and intensively through email, telephone, and face-to-face meetings. The other two groups suffered operational delays, partially due to several regulations of the Health InsurancePortability and Accountability Act coming into effect, and were therefore less active during the first phase of requirements definition.

Our contextual inquiry sessions with the project participants at MSKCC, NYU, and the University of Puerto Rico provided a detailed picture of information management across participating institutions. In general, information was managed in a highly fragmented fashion and in several different computing environments/applications. Members typically worked on several computers (such as home, office, and laptop) and maintained project-related and other work-related information in several places (such as Yahoo Calendar [Yahoo! Inc., Sunnyvale, CA, USA], personal digital assistants, application programs such as Netscape Communicator [AOL Inc., Dulles, VA, USA], and corporate email servers). While all subjects used MS Windows, they did not all use the same applications for the same tasks (eg, Netscape Communicator and Outlook were both used for email). We observed several breakdowns in the way information was shared among individuals. For instance, group meetings were not always recorded in all personal calendars, creating scheduling and coordination problems. In Puerto Rico, the unreliability and limited bandwidth of network connections made it routinely necessary to work around these obstacles.

It is important to note that needs assessment and requirements definition extended (and, in the case of the Oral Cancer Center, are extending) throughout the centers’ funding period. Collaboratory staff closely monitored how the activities in their respective center evolved and continually evaluated opportunities for support through collaborative software.

### Tool Evaluation and Implementation

Table 2 provides a summary of representative collaborative requirements and the selected tools.

**Table 2:** Collaborative requirements, sample tasks, and corresponding collaborative tools for the centers

**Requirement**	**Center**		**Representative Tasks**	**Products Implemented**
	**HIV/AIDS**	**Oral Cancer**		
Provide a center-wide website	X	X*	publish reports, announcements, and member database launch collaborative applications	HTML PHP mySQL
Install and support collaboratory tools	X	X	install software applications remotely train end users one-on-one troubleshoot end user problems	Microsoft NetMeeting, Timbuktu Pro Microsoft Remote Desktop Connection (Oral Cancer Center only)
Train groups in use of collaboratory tools	X	X	introduce participants to tool concept and functionality practice using features of tools	Microsoft NetMeeting, Timbuktu Pro CentraNow, Genesys Web/telephone conferencing (Oral Cancer Center only)
Manage group meetings and associated information		X	schedule meetings record agenda and minutes	Groove Meetings tool
Conduct real-time, small group meeting	X	X	share textual and numerical data share analysis results (eg, SPSS files) view histology slides through telemicroscopy (HIV/AIDS Center only)	NetMeeting, Timbuktu Conferencing, Virtual PC (HIV only) Genesys Web/telephone conferencing (Oral Cancer Center only)
Manage study patient appointments		X	schedule patients for multiple clinical appointments	Groove Calendar tool
Access research database and study-related documents		X	manage research data in MS Access database co-author study protocols	Groove File Sharing tool
Broadcast seminars	X		Share prepublication data and study progress	Placeware Auditorium iVisit

^*^ Implementation of requirement suspended

#### HIV/AIDS Center

Based on the initial set of requirements, the HIV/AIDS Center implemented a comprehensive public/private website, several tools for synchronous collaboration, and Web conferencing for virtual seminars. The website offered progress reports about the research collaborations, descriptions of core services, and a searchable database of all existing members. The website was also used for administrative tasks (eg, registering members, making announcements of upcoming events, distributing applications for developmental grants, archiving center presentations, and providing help documents for the collaboratory tools), launching collaboratory applications for meetings and presentations, and evaluating the center’s activity (eg, collecting survey data, recording observations, and creating usage logs).

**Figure 1:**
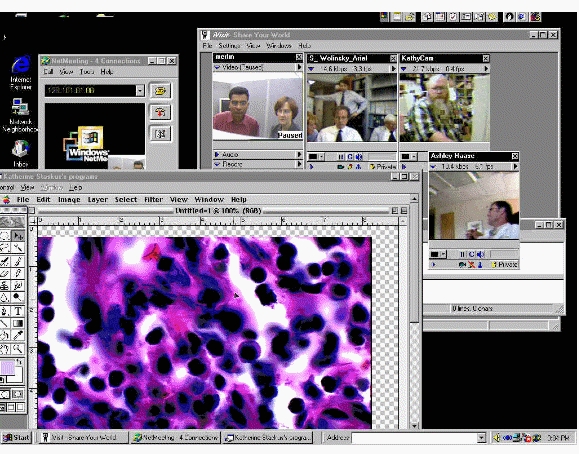
Screenshot of a virtual lab meeting in the HIV/AIDS collaboratory (the bottom left window displays the tissue sample being discussed by the participants shown in the four video feeds in the window at the right)

Point-to-point, real-time document, image, and equipment sharing was supported by Microsoft NetMeeting (Microsoft, Inc., Redmond, WA, USA). Because NetMeeting was not available on the Macintosh platform, Macintosh users used Timbuktu Conference (Netopia, Inc., Emeryville, CA, USA), and later, Virtual PC (Microsoft, Inc., Redmond, WA, USA). PlaceWare Auditorium (now owned by Microsoft, Inc. and marketed as Live Meeting), a Web-based presentation tool, was selected for virtual presentations. Since the quality of voice over IP (VoIP) connections was insufficient at the time of the HIV/AIDS project, we used telephones for audio during online sessions. On occasion, we used iVisit (iVisit, Santa Monica, CA, USA) to provide video in conjunction with NetMeeting and PlaceWare (Figure 1).

In order to implement these tools, the HIV/AIDS Collaboratory staff identified and trained a local support person at each of the four participating institutions. Although there was great variability in the expertise of the local support staff, the tools were successfully installed and tested before the project principal investigators used them. In addition, the regional nature of the HIV/AIDS collaboratory (including the fact that the collaboratory staff resided at one of the four member institutions) allowed collaboratory personnel to visit sites relatively easily when needed.

#### Oral Cancer Center

Just like the HIV/AIDS Center, the Oral Cancer Center initially focused on supporting communication and collaboration among members of the Oral Cancer Collaboratory as a whole. Based on the discussions with project personnel and the review of the Oral Cancer Collaboratory grant application, the collaboratory core personnel had a good understanding of the structure and workings of the center at large. On the other hand, gaining an understanding of each research project required a much longer period of time. In addition, because the individual research projects were just starting, their personnel, operational procedures, and infrastructure were still in development. We therefore developed a prototypical website that could function as the administrative “hub” for the center. After an initial period of high interest, it became clear that interaction among all center participants became less important than the increasingly intensive work on the research projects. We therefore suspended work on the center-wide website in order to focus on supporting each individual research group.

**Figure 2:**
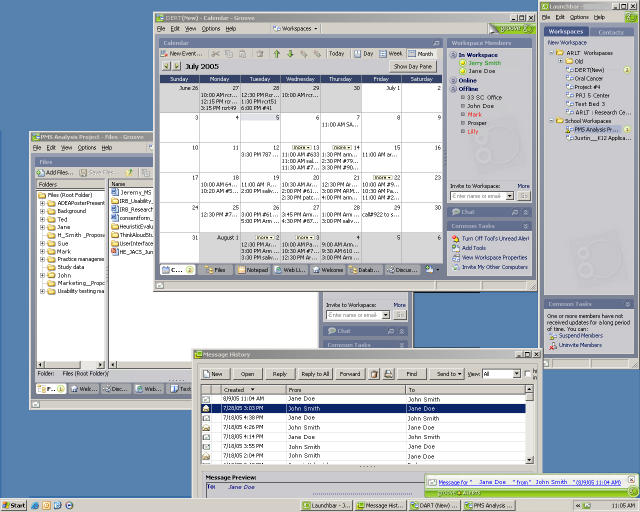
Screenshot of a desktop with two open Groove workspaces (the window on the right shows all available workspaces on this computer; the top window displays the calendar tool of the DERT(New) workspace; the background window shows the file sharing tool of the PMS Analysis Group workspace; the bottom center window contains the Groove Message History; messaging allows users to exchange short messages and can be configured to display notifications such as the one displayed in the lower right corner of the screen)

We evaluated several groupware applications, such as Lotus Notes (IBM, Armonk, NY, USA), eRoom (EMC Documentum, Pleasanton, CA, USA), Groove (Microsoft, Inc., Redmond, WA, USA), and Hyperwave (Hyperwave AG, München, Germany). We chose Groove (Figure 2) for a pilot implementation with the MSKCC/NYU research group for several reasons. Groove is secure, peer-to-peer collaborative software that integrates a wide variety of collaborative tools (such as file sharing, threaded discussions, Web links, document review, and calendar) into a single workspace (see Figure 2). An administrator can choose which tools are available in a particular workspace and therefore match the feature set to group requirements. Groove is relatively well integrated with the MS Office suite (Microsoft, Inc., Redmond, WA, USA), the application environment used by all of the Oral Cancer Center members. Due to its peer-to-peer architecture, Groove’s administrative overhead is much lower than that of some server-based applications (eg, Lotus Notes). Using NetMeeting, we trained the study personnel remotely in Groove functions relevant to their project tasks. The shared file area was the repository for patient data, and both MSKCC and NYU accessed and modified the same database. The shared calendar served as a tool to record past, current, and future clinical appointments for study participants. Groove’s meeting tool was intended to provide a facility for organizing and recording meetings. A member of the collaboratory core managed the group workspace in Groove, monitored feature usage, and took weekly snapshots of the workspace.

In Year 3, we evaluated Web conferencing software applications for use in joint project meetings for research groups, as well as for monthly updates of project and core principal investigators, and NIDCR personnel. We evaluated and pilot-tested CentraNow (Centra, Inc., Lexington, MA, USA), Elluminate (Elluminate, Inc., Calgary, AB, Canada) and Genesys Meeting Center (Genesys Conferencing, Inc., Reston, VA, USA). All three tools adequately satisfied our synchronous collaboration requirements, which included the ability to present PowerPoint slides, share applications, manage participation (eg, turn-taking for question-and-answer periods), and record online sessions. However, only Genesys could work through a firewall configured with standard ports, and thus was the default choice. To date, the research group for cancer screening and research subject participation by minorities has used Genesys for collaborating on survey data analysis.

In contrast to the HIV/AIDS Center, local support for collaboratory tools was problematic in the Oral Cancer Center. Since the collaboratory personnel were not co-located with any of the project principal investigators or research groups, they often had to interact directly with the principal investigators and research personnel. At some institutions, local IT support was not available; at others, IT support staff did not consider supporting the collaboratory tools as falling within their purview. Support issues frequently had to be solved remotely, often through screen-sharing tools. Occasionally, we provided support on-site during visits to member institutions.

### Collaboratory Evaluation

#### HIV/AIDS Center

Membership in the HIV/AIDS Center was open to anyone engaged in AIDS and AIDS-related research at the four participating institutions. By Year 4, there were 117 registered members of the HIV/AIDS Collaboratory (ranging from 16 to 42 members per site), representing significant growth in membership from the approximate 11 scientists involved in writing the center grant application. During the total funding period, the center sponsored the development of seven major research studies and funded pilot projects for nine junior-level scientists. The research portfolio of the center members increased by 64% in a period when the overall NIH budget for HIV/AIDS increased by 33%.

##### Virtual Lab Meetings

In the HIV/AIDS Center, a series of virtual lab meetings was established after the initial technical demonstration in the third quarter of Year 1. In the first six months of operation, the collaboratory was used seven times for virtual lab meetings, a rate of about once per month. The average attendance was 4.6 principal investigators (range 3-6) located at three to five computers spread over three of the sites. The principal investigators were typically joined by a number of members from their labs, as well as occasional guests, including NIH administrators, scientific advisory board members, and members of the press.

The scientists valued the virtual lab meetings for the ability to have real-time discussion accompanied by a shared view of a screen and a shared pointer. Specifically, the scientists discussed tissue sample images that were broadcast from a microscope located at one of the sites (see Figure 1), other summary patient data represented as graphics, research protocols, and co-authored documents. The tissue samples and patient data were gathered at several sites, but the expertise for analyzing these samples was located at only one site. As a novel form of collaboration for these scientists, viewing the data together gave them the opportunity to see the data collected at all sites, discuss analyses in real time and, for the tissue images, in the presence of the pathology expert. The scientists also used these meetings to initiate joint studies. The director of the center characterized this as a change from “little science to big science.” By bringing in members of their lab groups to these meetings, senior scientists made their scientific practice more accessible to the junior members of their research teams [22,23].

After the initial use of NetMeeting in the first six months after collaboratory deployment, larger group meetings occurred with less frequency. Specifically, there were five meetings over a 16-month period, organized when individuals generated research results that they wanted to share. However, more one-on-one use of the collaboratory tools emerged to support specific research projects, representing several new cross-site collaborations between pairs of scientists who had not worked together before the center grant began.

##### Virtual Seminars

The method of broadcasting seminars using the PlaceWare Auditorium software combined with a conference call was also used as a mechanism for sharing pre-published data among the center members. The first virtual seminar occurred at the beginning of Year 2 of the grant. In total, there were nine seminars, four in Year 2 and five in Year 3. Two HIV/AIDS Center members and seven speakers from outside the center presented these seminars. There were an average of 13 computers (range 5-19) logged into each presentation, located at three to four sites. This figure greatly underestimates the number of participants because people were typically assembled in groups around monitors and projection screens. A more accurate picture of seminar participation is derived from a survey administered to the full membership, showing that 73% of HIV/AIDS Collaboratory members who responded to the survey attended at least one, and on average three, virtual seminars. The primary reason for nonattendance was scheduling conflicts (78% of survey respondents), and only 5% reported not attending due to technical difficulties.

The collaboratory personnel in the HIV/AIDS Center attempted to introduce asynchronous data sharing (such as file sharing) to support group work. This effort was not successful, however, because the application deployed, Docushare (Microsoft, Inc., Redmond, WA, USA), was agnostic about content, and the scientists primarily wanted to use a common clinical database.

#### Oral Cancer Center

##### Shared Workspaces

The Personalized Risk Feedback in Dental Clinic Smokers study performed by MSKCC and NYU offered multiple opportunities for collaboratory support. A utilization analysis of the Groove workspace after 20 months of use (Table 3) showed that group members used the workspace to collaborate on files and to coordinate clinical appointments. After rapid initial growth, the increase in Word and Excel files leveled off. Recorded patient appointments grew at a steady pace because the clinical personnel used them to closely coordinate their day-to-day work. The group discontinued its use of the meeting tool to organize and record meetings because it did not integrate with the calendar tool in Groove or with the group members’ personal calendars. The number of tools used within Groove changed periodically as group members explored new tools and adopted only those that provided value. The number of members in the workspace fluctuated in the first five months, mainly because of technical problems (which led two senior group members to discontinue their use of Groove) and staff turnover. Subsequent to the pilot implementation with the MSKCC/NYU research group, the implementation of Groove with two other research groups is now in progress.

**Table 3:** Summary analysis of the use of the Groove workspace in the Personalized Risk Feedback in Dental Clinic Smokers study (all numbers represent the total number of objects at the time the workspace snapshot was taken)

**Year**	**Month**	**Number of Members**	**Word Files**	**Excel Files**	**Patient Appointments**	**Other Appointments**	**Meetings**	**Tools Used**
2003	May	3	7	0	0	0	2	4
	June	5	14	0	15	0	4	4
	July	2	22	4	64	1	4	6
	August	7	41	10	106	9	5	7
	September	4	45	11	143	10	6	7
	October	6	46	11	228	14	7	7
	November	6	46	11	318	19	8	6
	December	6	52	11	419	25	8	6
2004	January	6	54	11	512	25	8	6
	February	6	60	13	582	25	8	6
	March	7	63	15	647	26	8	6
	April	7	64	15	661	28	8	6
	May	7	66	15	674	28	8	6
	June	6	65	15	685	30	8	6
	July	6	60	17	701	30	8	6
	August	6	62	17	712	30	8	6
	September	6	64	17	719	30	8	6
	October*	4	40	7	722	30	8	6
	November	7	42	7	725	30	8	6
	December	7	45	9	729	30	8	6

^*^ In October 2004, the loss of a password resulted in the temporary loss of members and Word and Excel files.

##### Web Conferencing

So far, the Cancer Screening and Research Subject Participation by Minorities project has used Genesys five times with six participants each for biweekly meetings. The group is working on analyzing a survey data set and has been using Genesys for sharing analysis strategies and results. To date, the hour-long meetings have typically included three activities. Technical startup, which includes the time until all attendees have logged into the meeting and are ready to participate, initially took between 15 and 20 minutes, and has declined to between 5 and 10 minutes. The time dedicated to discussing shared visual artifacts has increased from about 10 minutes in the first meeting to an average of about 30 minutes. Telephone conference–only phases, which address the analysis strategies, the work plan, and organizational matters, consume the remainder of the time. The lengthy technical startup phase is due in part to the low general computer literacy of some participants, limited facility with the Genesys client software, and software usability problems. However, the added value of Web conferencing outweighs the current technical drawbacks. The project director commented, “I don't care that it takes us ten minutes to connect—the tool still allows us to do something which we could not do otherwise.”

## Discussion

As this evaluation has shown, the two collaboratories described in this paper exhibited some similarities, but they also differed in fundamental ways both in terms of organizational issues and technical needs. Table 4 briefly summarizes those aspects.

**Table 4:** Comparative overview of the HIV/AIDS Center and the Oral Cancer Center, as well as their collaboratories

	**HIV/AIDS Center**	**Oral Cancer Center**
Membership	open	closed
Specification of research projects	loose, developmental	predefined
Collaboration emphasis	general, cross-site	individual project group
Need for		
instrument sharing	high	none
real-time collaboration	high	low
data sharing	emerged late	high
Central collaboratory support	1 research assistant, 75% of collaboratory PI	1 research assistant, 20% of collaboratory PI
Remote IT support	predefined and dedicated	sporadic
Computing platforms	MS Windows, Macintosh, UNIX	MS Windows, Macintosh

The open membership and developmental nature of the HIV/AIDS Center were the primary reasons for the collaboratory focus on enabling general, cross-site collaborations with the capability of both one-on-one and group interactions. In contrast, the Oral Cancer Center was initiated with a much more specific work plan, and, therefore, the collaboratory emphasis was on supporting group work within individual projects. Real-time collaboration in the HIV/AIDS Center used a rich array of tools, resulting in types of collaboration that would not have been possible otherwise (for instance, the real-time discussion of tissue samples among pathologists and clinicians). For the Oral Cancer Center, making sure that the information for working on a particular project was available and up-to-date was initially more important than real-time interaction between co-principal investigators. The need for real-time collaboration only emerged when the first project transitioned to data analysis and interpretation.

### Barriers to and Enablers of Collaboration

The comparison of collaboratories also identified several barriers and enablers that affected the outcomes of the respective implementations. These aspects should be addressed through further research. The barriers included the following:

- **Multiple computing platforms:** Cross-platform issues were more problematic in the HIV/AIDS Center (with MS Windows, Macintosh, and UNIX platforms) than in the Oral Cancer Center (MS Windows and Macintosh only), but the collaboratory staff of both centers had to use various workarounds (eg, Virtual PC on the Macintosh) to allow certain members to participate.
- **Network infrastructure complexity:** A major hurdle for the Oral Cancer Center was to find Web conferencing software that worked with the firewall configurations of all participants. For the HIV/AIDS Center, firewalls were less of an issue as the local technical support staff could negotiate with systems administrators to provide access as needed.
- **Variable availability of local versus remote IT support:** The availability of local IT support personnel facilitated the installation and use of collaboratory tools in the HIV/AIDS Center. On the other hand, limited remote support was a major impediment for the Oral Cancer Center.
- **Low computer and collaborative software literacy:** Limited computer literacy with groupware tools hindered participants’ collaboratory adoption and use in both centers. While many scientists had some experience collaborating with distant colleagues, these collaborations typically relied on face-to-face meetings and email. Scientists in both centers needed strong incentives and low risk for adopting new ways of conducting their work.
- **Insufficient maturity of collaborative software:** Many collaborative software applications are relatively new products, and sometimes functional limitations, poor interface design, and bugs negatively affected the scientists’ perceptions of the value of these tools.
- **Lack of integration with existing application environments:** Collaborative tools should, as much as possible, integrate seamlessly with a user’s existing application environment [17]. This barrier was especially obvious for users of Groove in the Oral Cancer Center, as Groove provided stand-alone calendar and messaging functions which did not integrate with other applications. Similarly, in the HIV/AIDS Center, the need to use Virtual PC significantly decreased Macintosh users’ enthusiasm for several collaboratory applications.

Despite the problems described above, the comparison of the two collaboratories also identified several factors that promoted collaboratory adoption:

- **Collaboration incentives through funding mechanism:** In both centers, the funding mechanism promoted collaboration, albeit in two different forms. For the HIV/AIDS Center, funding was predicated on the development of projects and new collaborations, while for the Oral Cancer Center, it depended on adequate progress of predefined research projects.
- **Collaborative versus competitive relationship of researchers:** In neither center did competitive pressures among researchers inhibit their readiness to collaborate with other center members. The HIV/AIDS Center involved researchers with complementary expertise, and the Oral Cancer Center funded research projects with non-overlapping scientific questions. This structure ensured that each scientist’s own individual work did not threaten to “scoop” the work of a center colleague.
- **Leadership by example:** In the HIV/AIDS Center, the director led by example as he was an early adopter and one of the highest users of the collaboratory technology in his own center. In addition, several senior scientists not only quickly adopted the technology for their work within the center, but also began to use the tools for other collaborations as well. In the case of the Oral Cancer Center, the director actively sought out opportunities for the use of collaborative tools and strongly encouraged members to participate.
- **Tools matched to tasks:** In general, tools in both collaboratories were relatively well matched with project tasks. For instance, Groove provided the capability to reduce or expand the feature set of a workspace depending on the current needs of a project. On the other hand, in the HIV/AIDS Center, the general functionality of the document sharing application did not match the specific clinical needs, and the tool was therefore not adopted.
- **Technical progress:** During the lifetime of the HIV/AIDS Center, VoIP had not matured sufficiently to be a viable option for multicast audio of acceptable quality. By the start of the Oral Cancer Center, however, VoIP applications were feasible. Conversely, the bandwidth of Internet connections was sufficient to satisfy the performance demands of the collaboratory applications in the HIV/AIDS Center where the research sites were interconnected via Internet2. Members who suffered from “the last mile problem” [24] (eg, wiring in their buildings was not modern enough to capitalize on the bandwidth enabled by Internet2) often solved the problem by participating in the virtual meeting in a colleague’s office or in their lab located in a newer facility on campus.

### Judging Success of a Collaboratory

Applying the collaboratory model for distributed biomedical research will require further research on the factors related to successful application of the tools to the scientific activity. It is clear from the failure of the HIV/AIDS Center to be refunded that the presence of a collaboratory does not ensure collaboration between all participants. The success of this center in leveraging Wulf’s “collaboratory opportunity” [7] was judged differently by the NIH review panel and the center participants. A number of center members felt that their research benefited tremendously from the collaboratory and that they produced work with others with whom they would otherwise not have collaborated. An analysis of the scholarly output of 10 of the scientists who were the original principal investigators for the grant showed that 7 of the 8 new grants funded in the first three years of the center involved collaborators who had not previously been funded together. However, the NIH reviewers believed that the collaborations produced were not enough to merit the level of funding provided by a center. This question of what constitutes enough productivity to justify center-level funding is one that is very much unresolved at the current time and that has been articulated most recently by the National Science Foundation [25]. It is also unclear if the presence of a collaboratory increases the expectation for productivity that may lead to such centers being evaluated with more stringent success criteria than centers that employ more traditional mechanisms for collaboration.

### Conclusions

As collaboration technology continues to mature and become more commonplace in scientists’ everyday lives, the challenge will be to figure out how to integrate these tools into routine scientific practice in order to increase scientific efficiency and productivity. Disciplinary social norms will undoubtedly drive the pace and breadth of adoption of collaboratory tools [26]. For example, the rise in popularity of bioinformatics tools and the emphasis on exploiting cyber infrastructure for data archiving and management suggest that the capacity for sharing data is an important functionality for collaboration tools. However, without changes in the current reward structure for scientific advancement, incentives for contributing to and using such applications are unclear [27].

## Abbreviations

AIDS: acquired immunodeficiency syndrome
HIV: human immunodeficiency virus
IT: information technology
MSKCC: Memorial Sloan-Kettering Cancer Center
NIH: National Institutes of Health
NYU: New York University
PI: principal investigator
VoIP: voice over Internet protocol
